# The effectiveness of yoga to prevent diabetes mellitus type 2: A protocol for systematic review and meta-analysis

**DOI:** 10.1097/MD.0000000000014019

**Published:** 2019-01-18

**Authors:** Ramya Ramamoorthi, Daniel Gahreman, Simon Moss, Timothy Skinner

**Affiliations:** a College of Health and Human Sciences, Charles Darwin University, Darwin, Northern Territory, Australia; b Københavns Universitet, Institut for Psykologi, Center for Sundhed og Samfund, Københavns Universitet, Øster Farimagsgade, København, Denmark.

**Keywords:** meta-analyses, prediabetes, protocol, systematic review, yoga

## Abstract

**Background:**

Type 2 diabetes mellitus (T2DM) is becoming a leading problem worldwide. Emerging reports reveal alarming evidence of increasing prevalence of T2DM that has reached pandemic levels. Despite the significant incidence, there are limited reliable data resources and comprehensive systematic review and meta-analysis on the effects of yoga on people who are a prediabetic or high risk for developing T2DM.

**Objective:**

The objective of this protocol is to conduct a full-scale systematic review and meta-analyses on the effects of yoga on people who are prediabetes or high risk of developing T2DM.

**Methods:**

The articles enrolled in the study will be retrieved from the online databases between 2002 and the date the searches are executed. The searches will be repeated just before the final analyses and further relevant studies for inclusion. We will conduct a bibliographic search in databases: Medline/PubMed, Scopus, Cochrane Library, EBSCO, and IndMED using keywords including prediabetes state, high risk for diabetes, metabolic syndrome, and yoga. A defined search strategy will be implemented along with selection criteria to obtain full-text articles of relevant studies. This study protocol was prepared according to the Preferred Reporting Items for Systematic Reviews and Meta-Analysis for Protocols 2015 guidelines. There will be no language restrictions.

**Ethics and dissemination:**

The proposed study will be based on published studies and publicly available anonymized data without directly involving human participants and therefore requires neither formal human ethical review nor approval by a human research ethics committee. We published an outline of the protocol in the International Prospective Register of Systematic Reviews (PROSPERO) in 2018. We plan to disseminate the findings of this systematic review and meta-analysis through publication in a peer-reviewed journal and presentation at relevant conference proceedings. In addition, we believe the results of the systematic review will have implications for policy and practice. We will prepare policymaker summary using a validated format, disseminate through social media and email discussion groups.

**Review registration number:**

PROSPERO registration number CRD 42018106657

## Abstract 2

Supplemental Digital Content is available in the text

## 1 Introduction

Type 2 diabetes mellitus (T2DM) is one of the greatest public health challenges in today's world.^[1]^ The body becomes either resistant to insulin or gradually loses the ability to produce insulin.^[2]^ According to the World Health Organization, global report on diabetes incidence, an estimated 422 million people were found to be living with diabetes in 2014.^[3]^ International Diabetes Federation reported that it is estimated that there will be 629 million people with diabetes by the end of 2045, and diabetes-related health expenditure will exceed US$776 billion.^[4]^

The development and maintenance of T2DM are attributed to sedentary lifestyle,^[5]^ unhealthy diet, and psychologic stress. Psychologic stress has a strong correlation with both the risk factors^[6–8]^ and maintenance of the disease.^[9,10]^ Several acquired risk factors such as prediabetic state contribute to the development of T2DM apart from the genetic background.^[5,11]^

Many complementary and alternative practices are explored by people in both the prevention and treatment of diabetes.^[12,13]^ Yoga is one such Eastern practice that originated in India over 5000 years ago principally to develop mental faculties.^[14,15]^ Yoga advocates that a healthy body is a by-product of healthy mind.^[16,17]^ Most importantly, a growing body of research suggests that the practice of yoga may reduce insulin resistance syndrome and may attenuate signs, reduce complications, and improve the prognosis of diabetes.^[18–23]^ Also, studies have shown that the progression of diabetic condition from prediabetes could be either delayed or halted with regular physical activity,^[24–27]^ healthy diet,^[27]^ and effective stress management.^[28,29]^

It is proposed yoga intervenes T2DM by 2 proposed mechanisms downregulation of both the hypothalamic pituitary adrenal axis and the sympathetic nervous system.^[30–32]^

## 2 Rationale

### 2.1 What is the issue and how will our study address this?

Several studies in the prediabetic population show the effectiveness of yoga in reducing the risk of progression to diabetic state.^[33–36]^ There is not a single review to show the benefits of yoga in prediabetes. This will be the 1st systematic review that will show evidence that yoga significantly affects the prediabetic state and will summarize the results of these available studies. This study will announce the gaps in present research and will set directions for future research. The study will further provide evidence for people to adopt yoga practise as an attractive alternative to other forms of physical training especially for people who are discouraged by the perceived rigor of other exercises.

### 2.2 Review questions

The aim of our systematic review and meta-analysis protocol is to describe the methodologic approach for conducting a systematic review and meta-analysis to examine the effects of yoga on people who are prediabetic or high risk for developing T2DM.

The questions for this review are as follows:

1. Does yoga delay or prevent the progression of diabetes in prediabetic population?
2. What is the significance of yoga compared with exercise in a prediabetic population?
3. How much does the effect size of physiologic outcomes vary across studies and subgroups?

## 3 Methods

### 3.1 Search strategy and study selection

The proposed systematic review and meta-analysis will be performed according to the guidelines of Preferred Reporting Items for Systematic Review and Meta-Analysis (PRISMA) statement issued in 2015. The authors will consider the published studies explaining the effectives of yoga in prediabetic and metabolic syndrome with no restrictions on study participant's age, ethnicity, morbidity, and occupation. There will be no language restrictions. The authors will perform a literature search using 5 computerized English and Indian scientific electronic bibliographic databases: PubMed, Scopus, Cochrane Library, EBSCO, and IndMED. The search strategy will include only search terms related to “yoga” and “cardiovascular disease risk factors” and adapted for each database as necessary. Studies published between 2002 and the date the searches executed will be sought. The searches will be repeated just before the final analyses. Many searches in the proposed study will be undertaken to ensure the identification of eligible studies using one of the several search term combinations for the effectiveness of yoga on the high risk of diabetes or prediabetic population or metabolic syndrome (prediabetes state, high risk for diabetes, metabolic syndrome, and yoga). Keywords used were: “yoga [abstract],” “prediabetes [abstract],” and “glucose [text].” Keywords used included yoga + type 2 diabetes [in the title of the article], yoga + type II diabetes [in the title of the article], and exercise therapy + type 2 diabetes patients [in the title of the article].

### 3.2 Search strategy

A draft search strategy for the databases and search string that will be used to identify the studies describing effects of yoga in prediabetic and metabolic syndrome is shown in Table 1.

**Table 1:**
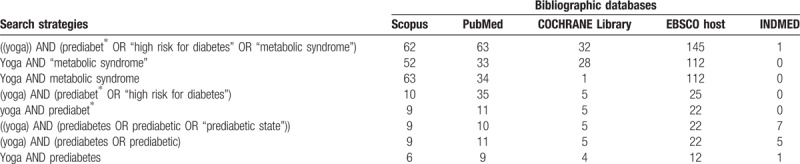
Search strings and databases used for the review and analysis.

### 3.3 Study selection criteria

#### 3.3.1 Inclusion criteria

1. Study examining yoga intervention (including at least one of asana, pranayama, meditation) to promote T2DM management and comparing yoga intervention with other usual care or physical exercise or nontherapeutic intervention.
2. Study that is randomized control trial, randomized cross-over studies, cluster-randomized trials, or quasi-experimental design will be included.
3. Studies evaluating the primary outcome measure-glycemic control, measured in both the intervention and control group conditions as well as other measures such as HbA1c, blood pressure, or fasting blood sugar, and lipid profile (triglycerides, high-, and low-density lipoprotein [HDL and LDL] cholesterol, systolic blood pressure [SBP], and diastolic blood pressure [DBP]) will be included.
4. Study participants must be prediabetic or designated as high risk for diabetes because of physiologic measures, and the outcomes must be reported specifically for each group

#### 3.3.2 Exclusion criteria

1. Studies will be excluded if participants were members of a specific age group, such as adolescents or geriatric age groups.
2. Studies will be excluded if participants were all in a transient state, such as pregnancy or menopause.
3. Studies will be excluded if the yoga intervention was modified to a dance program.
4. Conference proceedings, editorials, commentaries, and book chapters/book reviews will be excluded.

### 3.4 Data extraction and management

Two authors will be involved in data extraction and independently evaluate the published studies with the selection criteria, and corresponding authors will be contacted for missing information in the studies. Data will be extracted on study design and methods, demographic characteristics of study participants, as well as details of yoga interventions, control interventions, and outcome measures PRISMA guidelines will be used to prepare the data extraction form using MS Excel data extraction form. This form will be utilized to standardize the data collection process.

### 3.5 Selection process

**Figure 1:**
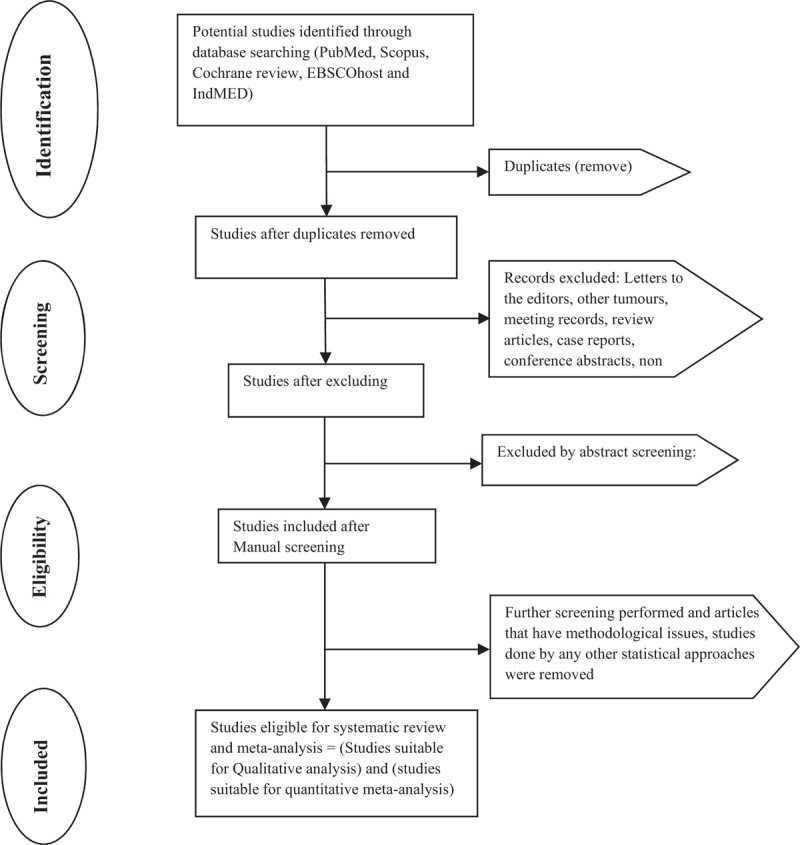
Flowchart of systematic review according to Preferred Reporting Items for Systematic Reviews and Meta-Analysis for Protocols statement.

The relevant titles and abstracts will be screened with the selection criteria and PRISMA guidelines for eligibility by the first author. Potential full-text eligible articles will be downloaded and reviewed independently by the authors. The corresponding author will perform a final review for the double check to recover any omitted articles in the analysis. The references of the selected articles will be imported into EndNote file to form an initial list of eligible studies following that duplicates will then be removed. All the authors will be involved in the selection process, and a file will be removed only when there is an agreement that it did not fulfill the eligibility criteria. Any discrepancies associated with selection of the studies will be resolved by mutual discussions involving the third reviewer. The entire selection process is illustrated in Figure 1.

### 3.6 Data items

The authors will extract 6 categories of data:

1. Bibliometric data (1st author, year of publication, country, a journal of publication, the study period).
2. Study design (a type of research, details of randomised control trial, randomized cross-over studies and cluster-randomized trials or quasi-experimental design and the validity of confirmative diagnosis and method of data collection).
3. Study participants characteristics (condition, age, gender, race, sample size, and sampling procedures).
4. Yoga interventions characteristics (yoga type: asana, pranayama, meditation, components, frequency, duration).
5. Control interventions characteristics (type: usual care or physical exercise or nontherapeutic intervention, frequency, duration).
6. Outcome measures: SBP, DBP; heart rate; respiratory rate; abdominal obesity (waist circumference, waist-hip ratio, index of central obesity); blood lipid levels (triglycerides, HDL, and LDL cholesterol); glycemic control (both the intervention and control group conditions, such as HbA1c, blood pressure, or fasting blood sugar).

### 3.7 Study outcomes

#### 3.7.1 Primary outcome

The primary outcome is to measure the glycemic control (HbA1c, fasting blood glucose [FBG], and postprandial glucose [PPBG]) in both the intervention and control group conditions.

#### 3.7.2 Secondary outcomes

The secondary outcomes are to measure other markers of diabetes management including triglycerides, HDL, LDL, SBP, DBP, body composition, and fasting cortisol.

### 3.8 Assessment of risk of bias

Risk of bias of included studies will be assessed using the Cochrane Risk of Bias Assessment tool that contains several items under 7 categories such as random sequence generation, allocation concealment, blinding of participants and investigators, the blindness of outcome assessments, incomplete outcome data, selective outcome reporting, and other biases. Based on the assessment, the studies will be evaluated as low, unclear, or high bias. The Jadad scale will be used to evaluate the quality of each trial where three domains in the scale cover Randomization (0–2 points), blinding (0–2 points), and dropouts and withdrawals (0–1 point). A trial with a score ≤2 indicates low quality while a score of ≥3 indicates high quality. Assessment of publication bias will be performed using funnel plots generated by Comprehensive Meta-Analysis (CMA) 3.0 software.

### 3.9 Data synthesis

Data will be synthesised into 3 different steps:

1. Step 1 will provide a descriptive overview (qualitative data synthesis or Systematic Review based on selection criteria and PRISMA guidelines).
2. Step 2 will provide a quantitative analysis of the characteristics of the selected studies (calculation of pooled estimates and meta-analysis). Meta-analysis will calculate pooled estimates of the study findings on the effectiveness of yoga interventions on the glycemic status of the prediabetic population.
3. Step 3 will examine the influence of study, participant, and outcome characteristics based on the difference in intervention among the prediabetic population by conducting subgroup and meta-regression analyses.

### 3.10 Meta-analysis

Meta-analyses on the effectiveness of yoga interventions on the glycemic status of the prediabetic population will be performed using CMA 3.0 for the obtained pooled estimates, standardized mean difference (SMD) and 95% confidence intervals from the included studies. Forest plots will be generated to show the pooled effect size of the study findings and with random-effects models of meta-analysis due to between-study heterogeneity into the model. Heterogeneity will be calculated using Cochrane *Q* test and *I*^2^ statistic. *Q* statistics will be estimated for each outcome and provides a test of the null hypothesis that all studies in the proposed meta-analysis share a common effect size. If all studies shared the same effect size, the expected value of *Q* would be equal to the degrees of freedom (the number of studies minus 1). *I*^2^ statistics informs what proportion of the observed variance reflects the difference in true effects sizes rather than sampling error. *Z*-statistic will be performed to assess heterogeneity. FBG, PPBG, TC, LDL-c, VLDL-c, HDL-c, and TG are reported as mg/dL, where studies reported as mmol/L a numerical conversion to mg/dL will be done. HbA1c is reported.

### 3.11 Publication bias

Publication bias of the included studies will be assessed using Egger bias indicator test, Orwin and Classic fail-safe *N* test, Begg and Mazumdar rank collection test, Duval and Tweedie trim and fill^[37]^ calculation. An inverted funnel plot will be constructed simultaneously alongside the forest plot, with the aid of SMD (SMD values used in the meta-analysis) and the standard error. The symmetrical funnel plots will indicate low risk, and asymmetrical funnel plots will indicate a high risk of publication bias.

### 3.12 Subgroup analyses

Subgroup analyses will be performed according to study, participant and outcome characteristics and methodologic factors if sufficient studies and retrieved data are identified and available. We plan to investigate specific subgroup analyses according to differences in intervention and key features of identified study participants such as condition, age, gender, race, sample size, and sampling procedures, follow-up, clinical setting, of prediabetic participants. Further, specific subgroup analyses will be performed based on the outcome measures such as blood pressure (systolic, diastolic); heart rate; respiratory rate; abdominal obesity (waist circumference, waist-hip ratio, index of central obesity); blood lipid levels (triglycerides, HDL, and LDL cholesterol); glycemic control (both the intervention and control group conditions, such as HbA1c, blood pressure, or fasting blood sugar) (if sufficient additional information is identified and available). Tables, flowchart, and figures will be plotted to depict the results appealingly.

## 4 Meta-regression

Prediabetic participant characteristics such as gender, methods of data collection, sample size, research quality, and sampling procedure will be evaluated. A random-effects model will be selected and assigned to weight for each study by calculating *R*^2^ with the quantity of the proposed variance. The heterogeneity of intervention associations with one or more study variables will be explained using meta-regression analysis.

## 5 Reporting of this review and its findings

The findings will be published as per PRISMA guidelines.^[38]^ A flowchart will be employed to outline the selection process (Fig. 1). Text description will be used to review the qualitative data of the included studies. Outputs of meta-analyses will be depicted in a forest plot. Publication bias will be represented in the inverted funnel plot. The search strategy will be provided in Supplement Table 1 (Supplemental Digital Content).

## Author contributions

**Conceptualization:** Ramya Ramamoorthi, Daniel Gahreman, Simon Moss, Timothy Skinner.

**Data curation:** Ramya Ramamoorthi, Daniel Gahreman, Simon Moss.

**Investigation:** Ramya Ramamoorthi.

**Methodology:** Ramya Ramamoorthi.

**Project administration:** Ramya Ramamoorthi.

**Resources:** Ramya Ramamoorthi.

**Supervision:** Daniel Gahreman, Simon Moss, Timothy Skinner.

**Validation:** Ramya Ramamoorthi, Daniel Gahreman.

**Visualization:** Ramya Ramamoorthi.

**Writing – original draft:** Ramya Ramamoorthi.

**Writing – review & editing:** Ramya Ramamoorthi, Daniel Gahreman, Simon Moss, Timothy Skinner.

## Supplementary Material

Supplemental Digital Content
