# Supplementary material for: The effectiveness of yoga to prevent diabetes mellitus type 2: A protocol for systematic review and meta-analysis
Source: Medicine (Baltimore). 2019 Jan 18;98(3):e14019. doi: 10.1097/MD.0000000000014019 (PMC6370061; doi:10.1097/MD.0000000000014019)
Supplement: Supplemental Digital Content [file medi-98-e14019-s001.doc]

**Supplement** Table 1: Search Strings and Databases used for the review and analysis.

| **Search Strategies** | **Bibliographic Databases** | | | | |
| --- | --- | --- | --- | --- | --- |
|  | **Scopus** | **PubMed** | **COCHRANE Library** | **EBSCO host** | **INDMED** |
| ((yoga)) AND (prediabet* OR "high risk for diabetes" OR "metabolic syndrome") | 62 | 63 | 32 | 145 | 1 |
| Yoga AND "metabolic syndrome" | 52 | 33 | 28 | 112 | 0 |
| Yoga AND metabolic syndrome | 63 | 34 | 1 | 112 | 0 |
| (yoga) AND (prediabet* OR "high risk for diabetes") | 10 | 35 | 5 | 25 | 0 |
| yoga AND prediabet* | 9 | 11 | 5 | 22 | 0 |
| ((yoga) AND (prediabetes OR prediabetic OR “prediabetic state”)) | 9 | 10 | 5 | 22 | 7 |
| (yoga) AND (prediabetes OR prediabetic) | 9 | 11 | 5 | 22 | 5 |
| Yoga AND prediabetes | 6 | 9 | 4 | 12 | 1 |
